# 维奈克拉联合阿扎胞苷治疗急性髓系白血病的近期疗效：单中心数据

**DOI:** 10.3760/cma.j.issn.0253-2727.2022.02.008

**Published:** 2022-02

**Authors:** 文静 于, 晋松 贾, 婧 王, 菲菲 唐, 立众 宫, 霄虹 刘, 晓璐 朱, 晓甦 赵, 晓军 黄, 浩 江

**Affiliations:** 北京大学人民医院，北京大学血液病研究所，国家血液系统疾病临床医学研究中心，北京 100044 Peking University People's Hospital, Peking University Institute of Hematology, National Clinical Research Center for Hematologic Disease, Beijing Key Laboratory of Hematopoietic Stem Cell Transplantation, Beijing 100044, China

**Keywords:** 白血病，髓系，急性, 维奈克拉, 阿扎胞苷, Leukemia, myeloid, acute, Venetoclax, Azacitidine

## Abstract

**目的:**

探究维奈克拉联合阿扎胞苷（Ven+AZA）在初治不适合强化疗（unfit）及难治（≥1次强化诱导化疗未缓解）/复发（R/R）急性髓系白血病（AML）患者中的近期疗效。

**方法:**

回顾性分析北京大学血液病研究所2019年6月1日至2021年5月31日接受Ven+AZA治疗（Ven 100 mg第1天、200 mg 第2天、400 mg第3～28天；阿扎胞苷75 mg/m^2^第1～7天）的初治unfit及R/R AML患者60例。分析各组完全缓解/血细胞计数未完全恢复的完全缓解（CR/CRi）率、微小残留病（MRD）转阴率及总体反应率（ORR）。

**结果:**

60例初治 unfit及R/R AML患者，中位年龄54（18～77）岁，男性33例（55.0％），中位随访时间4.8（1.4～26.3）个月。其中初治unfit患者24例（40.0％），R/R患者36例（60.0％）。初治unfit组及R/R组中位治疗疗程均为1（1～5）个。24例初治unfit患者（依据NCCN预后分层：低危8例、中危2例、高危14例）经第1疗程Ven+AZA治疗后，17例（70.8％）达CR/CRi，3例（12.5％）达部分缓解（PR），ORR为83.3％。其中9例患者接受第2疗程治疗，2例接受第3疗程治疗。17例CR/CRi患者中8例（47.1％）经2个疗程治疗后MRD转阴。36例R/R患者经第1疗程诱导治疗后，21例（58.3％）达CR/CRi（其中7例MRD转阴，占33.3％），3例（8.3％）PR，ORR为66.7％。R/R患者中12例治疗≥2个疗程，第2疗程治疗后无新增缓解患者，累计MRD转阴14例（66.7％）。R/R患者中低危组（CR至血液学复发≥18个月）12例，其余24例为高危组。低危R/R组1个疗程后10例（83.3％）达CR/CRi，CR/CRi率显著高于高危R/R组［45.8％（11/24），*P*＝0.031］。在60例患者中，13例伴IDH1/2突变及4例TP53阳性患者经1个疗程治疗后均达CR/CRi，18例NPM1突变阳性患者1个疗程CR/CRi率为77.8％，5例RUNX1-RUNX1T1伴c-kit D816患者（2例初诊，3例复发）均未获缓解。Ven+AZA作为诱导及再诱导治疗总体耐受性好。

**结论:**

Ven+AZA在初治unfit及R/R AML患者中均获得较高的治疗反应率，部分缓解患者可快速获得MRD转阴。其在伴NPM1、IDH1/2、TP53等基因突变的AML患者中疗效显著。

## Abstract (en)

**Objective:**

To explore the safety and short-term efficacy of venetoclax combined with azacitidine (Ven+AZA) in previously untreated patients unfit for standard chemotherapy and patients with relapsed/refractory (R/R) acute myeloid leukemia (AML) in China.

**Methods:**

A retrospective study was conducted in 60 previously untreated patients unfit for standard chemotherapy and patients with R/R AML who received Ven + AZA (venetoclax, 100 mg D1, 200 mg D2, 400 mg D3–28; azacitidine, 75 mg/m^2^ D1–7) at the Peking University Institute of Hematology from June 1, 2019 to May 31, 2021. The incidence of adverse events, complete remission (CR)/CR with incomplete hematological recovery (CRi) rate, objective remission rate (ORR), and minimal residual disease (MRD) status in patients with different risk stratification and gene subtypes were analyzed.

**Results:**

The median age of the patients was 54 (18–77) years, 33 (55.0％) were males, and the median follow-up time was 4.8 (1.4–26.3) months. Among the 60 patients, 24 (40.0％) were previously untreated patients unfit for standard chemotherapy, and 36 (60.0％) were R/R patients. The median mumber cycles of Ven+AZA in the two groups were both 1 (1–5). According to the prognostic risk stratification of the National Comprehensive Cancer Network, it was divided into 8 cases of favorable-risk, 2 cases of intermediate risk, and 14 cases of poor-risk. In previously untreated patients unfit for standard chemotherapy, after the first cycle of Ven+AZA, 17/24 (70.8％) cases achieved CR/CRi, 3/24 (12.5％) achieved partial remission (PR), and the ORR was 83.3％. Among them, nine patients received a second cycle chemotherapy and two received a third cycle. Among CR/CRi patients, 8/17 (47.1％) achieved MRD negativity after two cycles of therapy. In the R/R group, after the first cycle of Ven+AZA, 21/36 (58.3％) cases achieved CR/CRi (7/21 achieved MRD negativity), 3 achieved PR, and the ORR was 66.7％. Among R/R patients, 12 were treated for more than two cycles. There were no new CR/CRi patients after the second treatment cycle, and 14 cases (66.7％) achieved MRD negativity. According to the time from CR to hematological recurrence, the R/R group was divided into 12 cases in the favorable-risk group (CR to hematological recurrence ≥18 months) and 24 in the poor-risk group (CR to hematological recurrence <18 months, no remission after one cycle of therapy, and no remission after two or more cycles of therapy). Eleven of 24 (45.8％) cases achieved CR/CRi after one cycle of Ven+AZA in the poor-risk R/R group, and 10 of 12 (83.3％) achieved CR/CRi in the favorable-risk R/R group, which was significantly superior to the poor-risk group (*P*＝0.031). After one cycle of treatment, 13 patients with IDH1/2 mutations and 4 that were TP53-positive all achieved CR/CRi. The CR/CRi rate of 18 patients with NPM1 mutations was 77.8％. Five patients with RUNX1-RUNX1T1 combined with KIT D816 mutation (two initial diagnoses and three recurrences) had no remission. Ven+AZA was tolerable for AML patients.

**Conclusion:**

Ven+AZA has acceptable safety in previously untreated patients unfit for standard chemotherapy, patients with R/R AML can achieve a high response rate, and some patients can achieve MRD negativity. It is also effective in NPM1-, IDH1/IDH2-, and TP53-positive patients. The long-term efficacy remains to be observed.

急性髓系白血病（AML）异质性高，除部分低危患者可受益于强烈化疗，异基因造血干细胞移植是唯一具有治愈潜力的治疗方式。但对化疗不耐受及耐药的患者多数无接受造血干细胞移植的机会。维奈克拉（venetoclax，Ven）是一种口服的B细胞淋巴瘤2（Bcl-2）选择性抑制剂，目前多项研究及临床试验证实Ven联合去甲基化药物或低剂量阿糖胞苷（LDAC）在AML患者中可获得较高的治疗反应和无病生存率[1]–[2]。2020年10月美国食品药品管理局批准Ven与阿扎胞苷（AZA）、地西他滨或LDAC联合用于≥75岁或因合并症不能耐受强化诱导化疗的初治AML患者，且Ven+AZA方案于2020年被写入AML NCCN指南。2020年12月Ven在中国获批，并于2021年2月在中国上市。难治/复发（R/R）AML的挽救治疗中，VEN+AZA方案亦取得一定疗效[3]–[4]。目前国内尚缺乏对于VEN+AZA真实世界临床应用的报道。

## 病例与方法

1. 病例：纳入北京大学血液病研究所2019年6月1日至2021年5月31日接受Ven+AZA联合治疗且至少完成1个疗程的可评估疗效的AML患者共60例，分为初治不适合强化疗（unfit）组24例，R/R组36例。AML的诊断符合WHO标准[5]。收集患者相关资料，并进行随访。主要研究终点为完全缓解（CR）/血细胞计数未完全恢复的CR（CRi）率；次要研究终点包括总体反应率（ORR）、微小残留病（MRD）转阴率、3～4级不良事件（AE）。

2.治疗药物：药物来源：48例（80％）患者接受原研Ven治疗，其中18例为2021年2月前于中国香港自行购药，30例为2021年2月后中国大陆获批上市后使用；12例患者自行购买老挝Tongmeng Pharmaceutical&Food公司生产的Ventok（批号：12L0756/17），均为2021年2月前用药。

患者在第1疗程接受Ven剂量爬坡，第1天予Ven 100 mg，随后在3 d内逐步增至400 mg的目标剂量（100、200、400 mg）；之后继续给药，直到第28天，每日400 mg。从第2疗程开始，直接予每日400 mg口服。当合用CYP3A或P-gp抑制剂时（本研究中主要为伏立康唑），调整Ven剂量为100 mg每日口服。治疗方案中联合应用去甲基化药物AZA（75 mg/m^2^），在所有疗程的第1～7天通过皮下注射给药，每日1次。如出现难以控制的活动性感染、脏器损伤，治疗中止。

3. 定义：①初治unfit的标准：≥75岁或18～74岁且至少符合以下条件之一：美国东部肿瘤协作组（ECOG）体能状态评分为2～3分；严重的心、肺、肝或肾脏疾病；存在任何医师判断不适合接受强化化疗的合并症[6]–[7]。②难治：≥1个标准方案化疗后未达CR状态。③复发：CR的患者外周血中又出现白血病细胞或骨髓中原始细胞≥5％或出现新的病态造血或髓外白血病细胞浸润。④根据复发距离CR的时间，将复发患者分为低危组（CR至血液学复发≥18个月）及高危组（CR至血液学复发<18个月）。复发低危组定义为R/R低危组；复发高危组及难治患者定义为R/R高危组。⑤3～4级白细胞计数下降、血小板减少、贫血和发热性中性粒细胞减少根据美国癌症研究所4.0版不良事件（AE）通用术语标准评估。⑥终止治疗及中断治疗：终止治疗指因患者无法耐受、不良反应及治疗无效等原因不再实施治疗。中断治疗是指因不良反应等原因缩短本治疗疗程或推迟下一疗程治疗。⑦不易控制的活动性感染：抗炎治疗>2周评估仍不能接受标准化疗。⑧CR/CRi：CR定义为白血病的症状和体征消失，白细胞分类中无白血病细胞，骨髓中原始细胞<5％，无髓外白血病且中性粒细胞计数≥1.0×10^9^/L，PLT≥100×10^9^/L；CRi定义为白血病的症状和体征消失，白细胞分类中无白血病细胞，骨髓中原始细胞<5％，无髓外白血病且中性粒细胞计数<1.0×10^9^/L或PLT<100×10^9^/L。

4. 随访：采用电话、门诊随诊、医院病例登记系统等方式进行随访。随访截止时间为2021年6月30日。

5. 统计学处理：采用 SPSS24.0 软件进行数据分析。结果数据以中位数（范围）或数量（百分比）表示。计数资料比较采用*χ*^2^检验。*P*<0.05为差异有统计学意义。

## 结果

一、临床特征

截至2021年6月30日，接受Ven+AZA联合治疗的初治unfit及R/R AML患者中，至少完成一个联合治疗疗程的可评估患者共60例，其中21例（35.0％）因联用伏立康唑而调整Ven剂量为100 mg。本研究所纳入的60例患者中位年龄54（18～77）岁，男33例（55.0％），中位随访时间4.8（1.4～26.3）个月。24例（40.0％）为初治unfit患者，36例（60.0％）为R/R患者。初治unfit组及R/R组中位治疗疗程均为1（1～5）个。中位随访时间分别为2.9（1.5～13.2）个月和11.8（1.4～26.3）个月。初治unfit组和R/R组的临床特征详见表1。

**表1:** 接受维奈克拉联合阿扎胞苷治疗的初治不适合强化疗和难治/复发急性髓系白血病患者的临床特征

变量	初治不适合强化疗组（24例）	难治/复发组（36例）
年龄［岁，*M*（范围）］	59（22～77）	47（18～74）
性别［例（％）］		
男	15（62.5）	18（50.0）
女	9（37.5）	18（50.0）
诊断［例（％）］		
M_2_	14（58.3）	28（77.8）
M_4_	6（25.0）	4（11.1）
M_5_	4（16.7）	4（11.1）
NCCN危险分层［例（％）］		
低危组	8（33.3）	18（50.0）
中危组	2（8.3）	7（19.4）
高危组	14（58.3）	11（30.6）
按复发时间分层［例（％）］		
低危^a^	NA	12（33.3）
高危^b^	NA	24（66.7）
特殊基因标志［例（％）］		
NPM1^+^	3（12.5）	15（41.7）
IDH1/IDH2^+^	2（8.3）	11（30.6）
TP53^+^	3（12.5）	1（2.8）
FLT3-ITD^high^	2（8.3）	3（8.3）
RUNX1-RUNX1T1	4（16.7）	3（8.3）
RUNX1-RUNX1T1伴c-kit D816	2（8.3）	3（8.3）
MLL合并EVI1高表达^c^	3（12.5）	4（11.1）
不适合强化疗原因［例（％）］		
年龄≥75岁	2（8.3）	NA
活动性感染	16（66.7）	NA
脏器功能不全	6（25.0）	NA

注：^a^ 完全缓解（CR）至血液学复发≥18个月；^b^ CR至血液学复发<18个月；^c^ 除外MLL-AF9；NA：不适用

二、疗效评估

1. 初治unfit患者共24例，其中2例>75岁，16例为诊断时合并活动性感染，6例存在脏器功能不全（4例肾功能不全、1例肝功能不全、1例心功能不全）。诱导治疗的中位用药时间28（14～28）d，其中3例（12.5％）未完成28 d的治疗，原因均为感染。

24例初治unfit患者中，依据NCCN预后分层标准，低危8例、中危2例、高危14例，经第1疗程Ven+AZA治疗后，17例（70.8％）达CR/CRi，3例（12.5％）达部分缓解（PR），ORR为83.3％。其中9例患者接受第2疗程治疗，2例接受第3疗程治疗。CR/CRi患者中8例（47.1％）经2个疗程治疗后MRD转阴。24例初治unfit患者中，有4例患者经Ven+AZA诱导达CR/CRi后，unfit因素（不易控制的活动性感染）消除，后衔接标准化疗巩固治疗，经1～2个疗程巩固化疗后MRD转阴。

2. R/R组的诱导治疗中位用药时间为28（14～28）d，总CR/CRi率58.3％，ORR为66.7％。其中11例（30.1％）未完成28 d的治疗，主要原因为感染及重度骨髓抑制。低危R/R组1个疗程后10例（83.3％）达CR/CRi，高危R/R组1个疗程后11例（45.8％）达CR/CRi，差异有统计学意义（*P*＝0.031）。

在复发低危组患者中，9例为初诊时NPM1阳性，其中3例合并FLT3-ITD/FLT3-TKD突变，4例合并IDH1/IDH2突变，接受Ven+AZA中位疗程数为3（1～5）个。1个疗程后8例达CR/CRi，达CR/CRi所需中位疗程数为4（1～5）个；CR患者中，6例MRD转阴，MRD转阴时中位疗程数2（1～2）个。复发患者中3例为RUNX1-RUNX1T1阳性合并C-kit D816突变，均未达CR/CRi。

3. 不同基因亚型疗效分析：①NPM1突变阳性患者共18例，接受Ven+AZA中位疗程数为2（1～5）个。1个疗程CR/CRi率77.8％（14/18），后续治疗未增加达CR/CRi患者。CR/CRi患者中，MRD转阴率为57.1％（8/14），MRD转阴时中位疗程数2（1～2）个。②IDH1/IDH2突变阳性患者共13例，接受Ven+AZA中位疗程数为2（1～5）个。1个疗程CR/CRi率100％。CR/CRi患者中，MRD转阴率为69.2％（9/13），MRD转阴时中位疗程数2（1～2）个。③FLT3-ITD/FLT3-TKD阳性共5例，接受Ven+AZA中位疗程数为1（1～2）个。1个疗程CR/CRi 3例，1个疗程未获CR/CRi患者未行第2疗程治疗。CR/CRi患者中，MRD转阴1例。④TP53阳性患者共4例，其中初治unfit 3例，有2例为复杂核型，1例为正常核型，3例初治unfit患者突变位点分别为NM_000546:c.577C>T（p.H193Y）Exon 6，NM_000546:c.577C>T（p.H193Y）Exon 6，NM_000546:c.536A>G（p.H179R）Exon 5，VAF值分别为86.1％、92.2％、84.4％；难治1例，伴复杂核型，其突变位点为NM_000546:c.584T>C（p.I195T）Exon6，VAF值91.9％。接受Ven+AZA中位疗程数为2（1～2）个。1个疗程后均达CR/CRi。CR患者均MRD转阴，MRD转阴时中位疗程数1（1～2）个。其中3例初治unfit患者在1个疗程后均达到MRD阴性的CR。⑤RUNX1-RUNX1T1伴c-kit D816突变患者共5例，接受Ven+AZA疗程数均为1个，且均未达CR/CRi。⑥11q23合并EVI1高表达患者共7例，接受Ven+AZA中位疗程数为1（1～2）个。1个疗程CR/CRi 3例，后续治疗未增加达CR/CRi患者。CR/CRi患者中，MRD均未转阴。3例患者达PR，其中1例经第2疗程治疗后进展为NR，1例换用CAG方案再诱导，1例患者直接衔接造血干细胞移植。⑦EVI1高表达伴t（3;3）患者共3例，接受Ven+AZA中位疗程数为1（1～2）个。1个疗程后均未达CR/CRi。疗效评估详见表2。

**表2:** 接受维奈克拉联合阿扎胞苷治疗的初治不适合强化疗和难治/复发急性髓系白血病患者疗效评估（例）

组别	初治不适合强化疗		难治/复发	
	例数	CR/CRi	例数	CR/CRi
总体	24	17	36	21
NCCN危险分层				
低危组	8	6	18^c^	10
中危组	2	1	7	6
高危组	14	10	11	5
难治/复发组危险分层				
低危^a^	NA	NA	12	10
高危^b^	NA	NA	24	11
复发高危组	NA	NA	9	1
1个疗程NR	NA	NA	6	4
≥2个疗程NR	NA	NA	9	6
特殊基因标志				
NPM1	3	3	15	11
IDH1/IDH2	2	2	11	11
TP53	3	3	1	1
FLT3-ITD^high^	2	2	3	1
RUNX1-RUNX1T1	4	2	3	0
RUNX1-RUNX1T1伴c-kit D816	2	0	3	0
MLL(除MLL-AF9)合并EVI1高表达	3	2	4	2

注：CR：完全缓解；CRi：血细胞计数未完全恢复的CR；NR：未缓解；NA：不适用；^a^CR至血液学复发≥18个月；^b^CR至血液学复发<18个月；^c^其中3例为RUNX1-RUNX1T1伴c-kit D816突变

三、安全性评估

1. 血液学AE评估：血液学AE发生率为100％（60/60），其中中性粒细胞减少伴发热的发生率为40.0％（24/60）。3～4级白细胞计数下降的发生率为86.7％（52/60），其中36例治疗前即有3～4级白细胞计数下降，因治疗所致3～4级白细胞计数下降发生率为26.7％（16/60）。3～4级贫血的发生率为71.7％（43/60），其中31例治疗前即有3～4级贫血，因治疗所致3～4级贫血发生率为20.0％（12/60）。3～4级血小板减少的发生率为76.7％（46/60），其中36例治疗前即有3～4级血小板减少，因治疗所致3～4级血小板减少发生率为16.7％（10/60）。

2. 非血液学AE评估：最常见的非血液学AE为感染，其次为胃肠道反应。感染主要包括肺炎（13.3％）和软组织感染（6.7％），均为3级以上AE。胃肠道反应主要包括恶心呕吐（15.0％）和腹泻（3.3％）。2例（3.3％）患者出现肝功能损伤；有3例（5.0％）患者出现肿瘤溶解综合征；1例（1.7％）患者出现眼底出血。AE汇总见表3。

**表3:** 第1疗程维奈克拉联合阿扎胞苷治疗中出现的不良事件（AE）［例（％）］

不良反应	任何级别AE	≥3级AE
所有AE	60（100.0）	36（60.0）
血液学AE	69（100.0）	25（41.7）
白细胞减少	35（58.3）	16（26.7）
血小板减少	17（28.3）	10（16.7）
中性粒细胞减少伴发热	24（40.0）	24（40.0）
贫血	17（28.3）	12（20.0）
非血液学AE	26（43.3）	18（30.0）
肺部感染	8（13.3）	8（13.3）
软组织感染	4（6.7）	4（6.7）
腹泻	2（3.3）	1（1.7）
恶心等胃肠道症状	9（15.0）	7（11.7）
心房颤动	2（3.3）	1（1.7）
肝功能损伤	2（3.3）	1（1.7）
肿瘤溶解综合征	3（5.0）	2（3.3）
眼底出血	1（1.7）	0（0）

## 讨论

Bcl-2是一种抗凋亡蛋白，通过特异性抑制Bcl-2蛋白和激活内源性线粒体凋亡途径引起肿瘤细胞快速凋亡[1]–[2]。既往研究表明Bcl-2家族蛋白在AML细胞中高表达且大多数AML干细胞依赖Bcl-2生存[8]–[10]。Ven是一种口服的Bcl-2选择性抑制剂，前期临床试验结果提示Ven联合去甲基化药物或LDAC治疗AML疗效显著。

既往对于年龄≥75岁老年初治AML患者，指南推荐仅给予去甲基化治疗及低剂量化疗，但该方案的有效率仅为10％～50％，中位总生存（OS）时间少于1年[4],[11]–[14]，预后差；对于较年轻的初治unfit AML患者，如伴活动性感染，临床中需活动性感染控制后才可接受强化诱导治疗，故部分患者将无化疗机会或早期化疗相关死亡率高。R/R AML预后很差，治疗选择也非常有限。传统化疗方案治疗的长期存活率仅为30％～40％，治愈率不足10％[15]。既往研究表明，应用传统联合化疗方案，R/R AML的CR/CRi率约为36％[16]–[17]。

选择性Bcl-2抑制剂Ven和去甲基化药物AZA的联合是近年AML治疗的重大进展之一[18]–[19]。Ven+AZA在国际临床试验中已证实在老年和初诊unfit AML患者中可获得良好疗效[19]–[21]，而对于R/R患者，小样本的回顾性研究也提示了Ven联合AZA CR/CRi率可达到32％～51％[22]–[23]。但目前尚缺乏国内AML患者治疗的数据。本研究回顾性分析单中心Ven+AZA治疗初治unfit及R/R AML患者的真实世界情况。

本研究中，有8例低危的初治unfit AML患者接受Ven+AZA诱导治疗，6例达CR/CRi，均为1个疗程即达CR/CRi，证明在低危初治AML患者中，Ven+AZA方案亦可获得较好疗效。R/R AML患者CR/CRi率可达58.3％，明显优于传统化疗。复发低危组患者中NPM1阳性患者经诱导缓解及巩固治疗后可再次达深度分子学缓解且长期维持，此类基因亚型患者具有良好的生存预期，Ven+AZA为该亚型患者提供了新的治疗方案，也提示我们可尝试将Ven作为此类患者长期维持治疗方案，但该结论还需进一步临床研究证实。

本研究也探究了常见AML基因亚型（TP53、NPM1、IDH1/IDH2、FLT3-ITD、CEBPA双突变、RUNX1-RUNX1T1）与Ven+去甲基化药物疗效的相关性，结果与既往研究相似[6]，特殊突变类型的亚组（伴NPM1、IDH突变、FLT3-ITD/TKD、TP53突变）是Ven+AZA的优效亚型[24]。而对于伴RUNX1-RUNX1T1阳性，尤其伴C-kit D816突变患者效果不佳。既往本中心的研究已证实此类患者预后较差[25]，而本研究中Ven+AZA治疗亦不能改善其不良预后。关于Ven的耐药机制目前仍不明确，既往研究显示可能与Bcl-2家族蛋白MCL-1以及Bcl-X_L_的上调等多种因素相关[26]–[27]，还需进一步研究。

Ven+AZA在初治unfit及R/R患者的诱导治疗中均出现AE，以血液学AE及肺部感染最常见，这与既往研究报道的安全性特征一致。而在血液学AE规范化管理方面，患者治疗疗程中常出现严重骨髓抑制，建议治疗中如出现此情况，2～3周可进行骨髓穿刺，根据细胞形态学及免疫分型评估肿瘤负荷，如骨髓细胞形态及免疫分型未见明显原始细胞，则考虑骨髓抑制4级血液学AE，可适当缩短疗程。

本研究有以下局限性：为回顾性分析且患者应用的疗程较少；随访时间相对较短，生存期的数据不足。我们将进一步开展前瞻性研究，扩大样本量，以进一步验证该方案的安全性和有效性。

Ven+AZA在AML患者中有良好的疗效和安全性，不仅可作为初治unfit AML患者的诱导治疗选择，明显改善老年AML患者预后，且对于年轻unfit及难治复发患者，可作为衔接异基因造血干细胞移植的桥梁。
